# Role of Dietary Antioxidants in p53-Mediated Cancer Chemoprevention and Tumor Suppression

**DOI:** 10.1155/2021/9924328

**Published:** 2021-06-26

**Authors:** J. P. Jose Merlin, H. P. Vasantha Rupasinghe, Graham Dellaire, Kieran Murphy

**Affiliations:** ^1^Department of Plant, Food, and Environmental Sciences, Faculty of Agriculture, Dalhousie University, Truro, NS, Canada; ^2^Department of Pathology, Faculty of Medicine, Dalhousie University, Halifax, NS, Canada; ^3^Faculty of Medicine, University of Toronto, Toronto, ON, Canada

## Abstract

Cancer arises through a complex interplay between genetic, behavioral, metabolic, and environmental factors that combined trigger cellular changes that over time promote malignancy. In terms of cancer prevention, behavioral interventions such as diet can promote genetic programs that may facilitate tumor suppression; and one of the key tumor suppressors responsible for initiating such programs is p53. The p53 protein is activated by various cellular events such as DNA damage, hypoxia, heat shock, and overexpression of oncogenes. Due to its role in cell fate decisions after DNA damage, regulatory pathways controlled by p53 help to maintain genome stability and thus “guard the genome” against mutations that cause cancer. Dietary intake of flavonoids, a C_15_ group of polyphenols, is known to inhibit cancer progression and assist DNA repair through p53-mediated mechanisms in human cells via their antioxidant activities. For example, quercetin arrests human cervical cancer cell growth by blocking the G_2_/M phase cell cycle and inducing mitochondrial apoptosis through a p53-dependent mechanism. Other polyphenols such as resveratrol upregulate p53 expression in several cancer cell lines by promoting p53 stability, which in colon cancer cells results in the activation of p53-mediated apoptosis. Finally, among vitamins, folic acid seems to play an important role in the chemoprevention of gastric carcinogenesis by enhancing gastric epithelial apoptosis in patients with premalignant lesions by significantly increased expression of p53. In this review, we discuss the role of these and other dietary antioxidants in p53-mediated cell signaling in relation to cancer chemoprevention and tumor suppression in normal and cancer cells.

## 1. Introduction

Cancer is a leading cause of mortality in the world, reporting 18.1 million new cases and 9.6 million deaths in 2018 [1]. National Institutes of Health of the US estimated 1.8 million new cancer patients in 2020, with 606,520 deaths in the US [2]. Conventional therapies for cancer include surgery, radiotherapy, chemotherapy, endocrine therapy, and targeted therapy [3, 4]. However, currently available therapies have serious limitations, including severe side effects and dose-limiting toxicities [5]. The common adverse effect of cancer treatments includes nausea, vomiting, peripheral neuropathy, pain, and insomnia, which reduce the quality of life [6]. Hence, the search for alternative therapeutic agents with better patient outcomes is necessary. Currently, epidemiological studies have revealed a strong association between the risk of developing cancer and the consumption of fruit and vegetables [7, 8].

Plants produce various defensive secondary metabolites such as polyphenols, isoprenoids, alkaloids, sulforaphanes, and certain vitamins and their precursors [9–11]. Most of these phytochemicals exert hormetic effects: at low concentrations, they act as antioxidants to scavenge free radicals, including reactive oxygen species (ROS), thus protecting cells from oxidative damage to proteins, lipids, DNA, and RNA [12]. Many studies have suggested that the regular intake of fruits, vegetables, and their products containing phytochemicals is effective in the prevention of various types of cancers [13–16]. Phytochemicals also exhibit anticancer activity through various signaling pathways, including the death receptor or extrinsic pathway, mitochondrial or intrinsic pathway, and perforin/granzyme apoptotic pathway [8, 17]. Polyphenols use the p53 signaling pathway to produce anticancer activity through apoptosis in various types of cancers [18, 19]. p53 regulates various biological processes, including metabolic pathways, aging, development, reprogramming, and reproduction [20, 21]. In this review, we are focusing on p53 regulation by various dietary antioxidants in relation to various cancers.

## 2. A Brief Overview of p53

In 1979, p53 protein was identified. SDS-PAGE analysis indicated that the protein has 53 kDa molecular mass, thus named p53 [22, 23]. In humans, the p53 gene is located on the short arm of chromosome 17 (17p13.1) [24]. p53 is well known as “the guardian of the genome” and is a powerful tumor suppressor, which comprises 393 amino acid residues and three main domains, namely, transcriptional activation domain (TAD), DNA-binding domain (DBD), and tetramerization (TET) domain. TAD recruits RNA polymerase and activates the DNA-reading machinery. DBD is rich in arginine/lysine residues, most prone to mutations, and binds to the specific regulatory sites present on the DNA response elements. The TET domain assembles the chains of other p53 monomers for oligomerization [25, 26].

## 3. Significance of p53

The main role of p53 is to control cell cycle progression, DNA repair, and apoptosis. In vertebrates, p53 has the ability to temporarily block the cell cycle and promote DNA repair. Under certain conditions, p53 can also induce senescence or promote apoptosis, thus providing mechanisms against the accumulation of potentially malignant or defective cells [27].

### 3.1. Apoptosis

**Figure 1:**
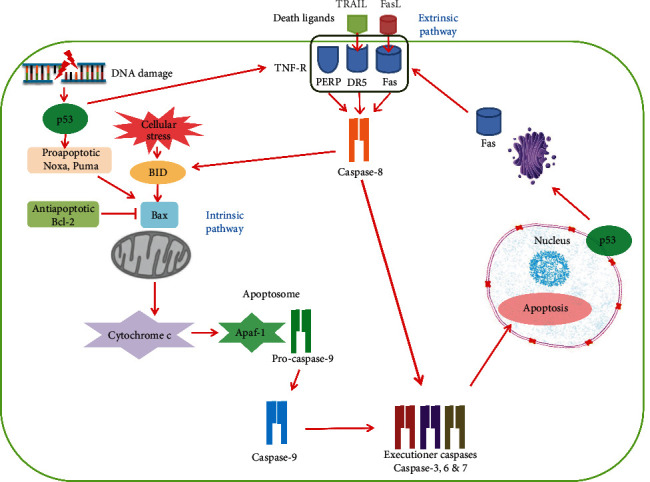
A schematic of the p53-mediated apoptotic pathways in a mammalian cell. The apoptotic pathways involve intrinsic and extrinsic pathways. p53 activates the extrinsic pathway through the induction of the tumor necrosis factor receptor (TNF-R) superfamily that contains apoptotic death domains such as Fas, DR5, and PERP. Overexpression of p53 enhances levels of Fas at the cell surface by promoting the trafficking of the Fas receptor from the Golgi. Fas is activated by its ligand FasL, and DR5 is initiated by the ligand TRAIL. TNF-R is induced by p53 in response to DNA damage, which also promotes cell death through the caspase-9-mediated intrinsic pathway. The intrinsic pathway is dominated by the Bcl-2 family, including Noxa, Puma, and Bax. Bax is activated by BID to increase the mitochondrial outer membrane permeability, allowing efflux of cytochrome c to cytosol. BID is activated by cellular ROS and also cleaved by caspase-8. BID is a crossregulator between the extrinsic and intrinsic pathways. The released cytochrome c and Apaf-1 form a complex with pro-caspase-9 named apoptosome in which caspase-9 is activated. Caspase-8 is activated by death domains of the extrinsic pathway and cleaves proenzyme to form caspase-3, caspase-6, and caspase-7, which affect cell viability and overall apoptosis induced by p53. *Abbreviations*: Apaf-1: apoptotic protease-activating factor 1; Bax: Bcl-2-associated X protein; Bcl-2: B-cell lymphoma 2; BID: BH3 domain-only death agonist protein; DR5: death receptor 5; Fas: Fas cell surface death receptor; FasL: Fas ligand; Noxa: damage protein, a proapoptotic BH3-containing protein; p53: tumor suppressor; PERP: p53 apoptosis effector related to PMP22; Puma: p53 upregulated modulator of apoptosis; TNF-R: tumor necrosis factor receptor; TRAIL: tumor necrosis factor-related apoptosis-inducing ligand.

Apoptosis is the cellular terminal process in the prevention of the proliferation of cells with anomalous DNA. In mammalian cells, p53 mediates apoptosis through transcriptional activation of proapoptotic target genes by two major apoptotic pathways: extrinsic pathway, mediated by death domain proteins belonging to the tumor necrosis factor receptor (TNF-R), and intrinsic pathway that monitors the B-cell lymphoma 2 (Bcl-2) family of proteins, which lead to cell death through the expression of caspases [28, 29]. The extrinsic apoptotic pathway is initiated by apoptotic genes such as Fas cell surface death receptor (Fas), death receptor 5 (DR5), and p53 apoptosis effector related to PMP22 (PERP) [30, 31]. Overexpression of p53 may increase the level of Fas at the cell surface by promoting the trafficking of the Fas receptor from the Golgi, which allows p53 to rapidly sensitize cells to Fas-induced apoptosis before the transcription-dependent effect operates [32]. Fas is activated by binding of its ligand FasL, which is expressed by T-cells [30]. DR5 is initiated by tumor necrosis factor- (TNF-) related apoptosis-inducing ligand (TRAIL), which promotes apoptosis through the caspase-8 pathway [33]. The intrinsic pathway involves the induction of the proapoptotic Bcl-2 family members Bcl-2-associated X protein (Bax), p53 upregulated modulator of apoptosis (Puma), and damage protein, a proapoptotic BH3-containing protein (Noxa) [33, 34]. The Bcl-2 family is activated by Bcl-2 homology-3 (BH3) domain-only death agonist protein (BID) to form channels in the mitochondrial membrane, allowing efflux of cytochrome c (Cyt c). BID exists in the cytoplasm as an inactive state. After exposure to TNF-R, BID is cleaved by caspase-8 and translocates to the mitochondrial membrane and activates Bax for apoptosome formation. BID is a link between the extrinsic and intrinsic pathways [30, 35]. p53 is also involved in the activation of the apoptosome via induction of Cyt c, apoptotic protease-activating factor 1 (Apaf-1) from mitochondria, and their formation of a complex with pro-caspase-9, which activates caspase-9 [33, 36]. Caspase-9 responds to changes in the mitochondrial perspective, whereas caspase-8 senses activation of death receptors. These initiator caspases cleave the proenzyme forms of the effector caspases to caspase-3, caspase-6, and caspase-7, which allows for the digestion of essential targets that affect cell viability (Figure 1) [37].

### 3.2. Senescence

The irreversible cell cycle arrest will lead to cellular senescence that can limit the expansion of mutations in cells [38]. Therefore, senescence is a way to prevent cancer. Apart from this, the depletion of p53 compromises the oncogene-induced senescence, and although molecular mechanisms are still elusive, several microRNAs and p53-targeted genes have been linked to senescence and depletion of p53 [39, 40]. The p53 protein acts both the prooxidant and antioxidant functions in relation to the intensity of the stress, which can contribute to tumor suppression [41]. p53 acts as a prooxidant in permitting the cells to repair damage and survive if stress is mild and also functions as an antioxidant to subsidize the overall action of apoptosis when stress is at an extreme level [19, 42].

### 3.3. Cell Cycle Arrest

Cyclins and cyclin-dependent kinases (CDKs) are the two major proteins that control cell cycle progression. The cell cycle is arrested at the G_2_/M phase during DNA damage, which prevents the propagation of mutagenic lesions to the daughter cells, and these transitions are monitored by p53 downregulatory p21 during the DNA damage [43]. The first evidence of participation of p53 in the cell cycle was confirmed by Kastan and colleagues in 1991, and they found the expression of GADD45, which was dependent on the presence of wild-type p53 by ionizing radiation [44]. DNA damage is also done by proteins such as GADD45 induced by p53. p53 plays an important role in mitotic cell division by regulating the function of centrosomes by blocking the mitotic spindle assembly using nocodazole, which leads to the activation of p53 by ataxia telangiectasia-mutated protein kinase (ATM), the key initiator of the DNA damage repair (DDR) pathway [33, 45].

### 3.4. DNA Repair

The endpoint of the DDR cascade is DNA repair. Various internal factors that cause DNA damage are ROS, free radicals, certain cellular metabolites, and various external factors such as UV light, certain drugs, and heavy metals. All these factors lead to the moderation of nucleotide bases of DNA, resulting in DNA strand breakage [33, 46]. p53 is active in regulating DNA damage, and its key function is to promote nonhomologous end joining (NHEJ) and suppress homologous recombination (HR). The two major pathways involved in DNA repair are NHEJ and HR. Certainly, the mechanism of these two processes of DNA repair may differ. HR is an error-free process, while NHEJ is very fast in DNA repair. p53 phosphorylates a series of effector molecules such as rap1-interacting factor 1 (RIF1) and pax transactivation domain-interacting protein (PTIP), prevents resection of DNA 5′ ends, and promotes NHEJ [47–49]. DNA damage results in DNA repair by growth arrest and the DNA damage-inducible gene (GADD45) family that is composed of GADD45*α*, GADD45*β*, and GADD45*γ* nuclear proteins, G_1_ arrest by p21, and also results in apoptosis by apoptotic genes. p53 can also induce GADD45, enhancing nucleotide excision repair (NER) to reduce DNA damage and UV photoproducts [50]. p53 also exerts its regulation of DNA function by base excision repair (BER). BER is used to remove the chemically modified bases on the basic (apurinic or apyrimidinic (AP)) nucleotide. The basic nucleotide site is removed by pyramidic endonuclease, resulting in a breach filled by DNA polymerase and a ligase [51]. p53 also activates more DNA damage repair genes to maintain genome stability and regulates the centrosome via duplication and replication. The lack of p53 can be associated with an increased level of genomic agitation, which may be the reason for causing oncogenic progression [52–54].

## 4. Regulation of p53

Excessive activity of p53 is highly harmful to cell survival and normal growth conditions; thus, the p53 protein level is regulated by ubiquitination and degradation, which is mediated mainly by the E3 ubiquitin ligase mouse double minute 2 homolog (MDM2) [55]. Cells are exposed to various stress signals, including oxidative stress, oncogene activation, telomere shortening, and hypoxia, which leads to p53 activation by inducing MDM2 [56]. The regulation of MDM2 and its close homolog—double minute X human homolog (MDMX)—is achieved by negative and positive regulators, which creates a feedback mechanism. The primary mechanism for suppressing p53 is its negative regulator, MDM2 [33, 57]. MDM2 is induced by p53, and p53 triggers are self-destruction through a negative feedback loop. It has been found that an increased level of MDM2 inactivates the functions of p53, such as apoptosis and cell cycle arrest. MDM2 and p53 form the autoregulatory feedback loop to control cellular activity. MDM2 acts as an E3 ubiquitin ligase and is responsible for the ubiquitination and degradation of p53 [33, 58]. Several reviews broadly discuss the roles of the p53-MDM2 pathway in the initiation, progression, and metastasis of human cancers [59, 60]. Therefore, p53 and MDM2 are critical for controlling their expression levels in both the normal and cancer cells [61, 62].

### 4.1. Normal Cells

The p53 protein is very low in normal cells due to continuous ubiquitination by MDM2 and MDMX [63]. p53 is controlled by MDM2 and MDMX in normal cells, and this control is necessary for the cells to turn on p53 promptly and effectively utilize its remarkable antioncogenic power to maintain a cancer-free environment [56, 64]. p53 accumulated in normal cells activates the expression of MDM2 in the downstream signaling pathway. MDM2 binds with the transcriptional activation domain of p53 to form the p53-MDM2 complex, which inhibits the transcriptional activity of p53 and creates negative feedback to regulate the pathway [24, 65]. The DNA damage signaling pathway can activate p53 by phosphorylating MDM2 and MDMX and blocks the feedback regulations on this protein through the stress-induced kinase cascade, such as ATM, ATM-Rad3-related protein kinase (ATR), checkpoint kinase 1 (Chk1), and checkpoint kinase 2 (Chk2) [56, 66–68]. p53 protein is activated primarily through posttranslational modifications, which leads to the increase of p53 protein half-life; therefore, p53 protein is accumulated in cells [69]. There are more than 100 target genes of the p53 protein, which have been identified and involved in the regulation of apoptosis by apoptotic genes, such as Bax, Fax, Noxa, DR5, PERP, and Puma, and in cell cycle arrest by genes including p21, GADD45, Yippee-like 3 (Ypel3), and promyelocytic leukemia protein (Pml) [70]. These genes are associated with the regulation of p53, including cellular signaling, regulation of the extracellular matrix, and cellular structure [71]. The activation of p53 is regulated by several modifications, such as acetylation, methylation, ubiquitination, and phosphorylation [26].

### 4.2. Cancer Cells

The p53 gene is classified as a wild type and mutant type. It has been suggested that more than 50% of all malignant tumors have mutations in the p53 gene [65]. The mutated p53 gene can induce and promote tumorigenesis than the wild-type p53; tumor suppressor genes can transactivate and perform broad-spectrum tumor suppression [72]. Loss of p53 function is strongly associated with cancer development across a wide range of organs [73, 74]. Animals bearing mutant p53 have severely compromised transcriptional activity, making them prone to tumor development [75]. Broad mutation search suggested that more than half of the human cancers possess mutations of p53 [76]. About 95% of p53 mutations were noticeable within the genomic region, encoding the DNA-binding domain [77]. About 30% of mutations occur in six amino acid residues, which are most frequently mutated in human cancers, including Arg175, Gly245, Arg248, Arg249, Arg273, and Arg282 [78]. These mutations are initiated within the DNA-binding domain of p53 and subsequently disrupt the native conformation of the protein. Therefore, the mutant p53 is defective in the sequence-specific transcriptional activation dependent on the wild-type p53-binding consensus element. Moreover, the mutant p53 shows a dominant-negative behavior towards the wild-type p53 through the formation of heterotetramer with wild-type p53 and has the oncogenic potential [79, 80].

Mutant p53 protein interacts with several transcription factors, such as nuclear factor Y (NF-Y), E2F transcription factors (E2Fs), nuclear factor NF-kappa B p65 (NF-*κ*Bp65), nuclear factor NF-kappa B p50 (NF-*κ*Bp50), sterol regulatory element-binding protein (SREBP), Yes-associated protein (YAP), vitamin D receptor (VDR), and nuclear factor erythroid 2-related factor 2 (Nrf2), which modulates the expression of diverse sets of genes. Mutant p53 proteins lose their ability to activate p53 target genes, and some mutants exert transdominant repression over the wild type [81–84]. The gathering of mutant p53 protein plays an important role in oncogenic activity. The mutant p53 protein is often stabilized in cancer cells, whereas wild-type p53 has a short half-life in both normal tissues and cancer cells [85]. Similar to wild-type p53, mutant p53 degradation is also proteasome-mediated and promoted by the E3 ubiquitin ligases MDM2 and chromatin immunoprecipitation (ChIP) [86]. Still, the mutant p53 becomes stabilized and activated in response to tumor-related stress conditions, similar to wild-type p53 [87]. The accumulating evidence revealed that certain cancer-derived mutant forms of p53 transactivate various target genes, including multiple drug resistance gene 1 (MDR1), c-myc, proliferating cell nuclear antigen (PCNA), interleukin-6 (IL-6), insulin-like growth factor 1 (IGF-1), fibroblast growth factor (FGF), epidermal growth factor receptor (EGFR), asparagine synthetase (ASNS), and telomerase reverse transcriptase (TERT) [76, 88, 89]. Therefore, cancer-derived mutant p53 promotes adaptive responses to growth-promoting and oncogenic genes, thereby leading to the progression of aggressive cancers [90].

## 5. Regulatory Mechanism of the p53 Signaling Pathway

**Figure 2:**
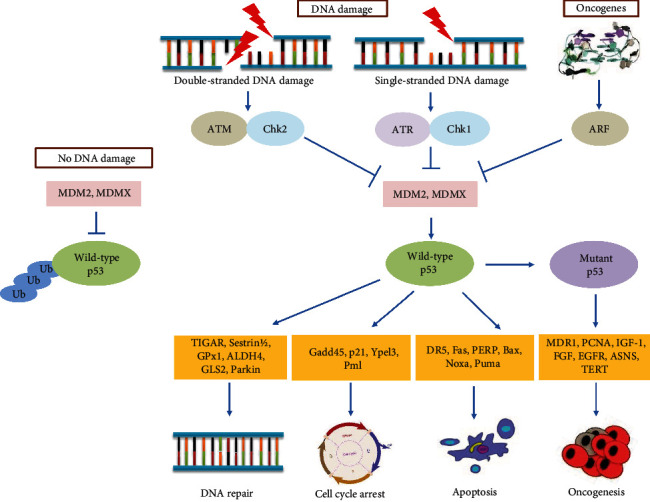
DNA damage and oncogenic signaling active the p53 tumor suppressor to maintain genomic stability and prevent transformation. Both the double-stranded DNA break and the single-stranded DNA break trigger the activation of p53 through ATM and ATR, respectively. Chk1 and Chk2 are selectively phosphorylated and activated by ATR and ATM, respectively. Oncogenic signaling produces the ARF tumor suppressor to inhibit the expression of MDM2 and MDMX. MDM2 and MDMX are the two negative regulators that function as an E3 ubiquitin ligase, which recognizes the N-terminal transcriptional activation domain (TAD) of p53, and as an inhibitor of p53 transcriptional activation. Therefore, under no stress or no DNA damage, p53 is kept inactive by the negative regulation by MDM2 and MDMX. Once activated, p53 induces antioxidant genes such as TIGAR, sestrin 1/2, GPx1, ALDH4, GLS2, and Parkin to repair the DNA. Growth arrest and DNA damage-inducible proteins such as GADD45, p21, Ypel3, and Pml are induced by p53 to cell cycle arrest and apoptotic genes, including DR5, Fas, PERP, Bcl-2, Bax, Noxa, and Puma for p53-mediated apoptosis. If the p53 gene is damaged, tumor suppression is severely reduced. Mutant p53 transactivates several genes, including MDR1, c-myc, PCNA, IL-6, IGF-1, FGF, EGFR, ASNS, and TERT. Mutant p53 promotes oncogenic genes, which lead to the progression of aggressive cancers. *Abbreviations*: ALDH4: aldehyde dehydrogenases; ARF: alternate reading frame; ASNS: asparagine synthetase; ATM: ataxia telangiectasia-mutated protein kinase; ATR: ATM-Rad3-related protein kinase; Bax: Bcl-2-associated X protein; Bcl-2: B-cell lymphoma 2; Chk1: checkpoint kinase 1; Chk2: checkpoint kinase 2; DR5: death receptor 5; EGFR: epidermal growth factor receptor; Fas: Fas cell surface death receptor; FGF: fibroblast growth factor; GADD45: growth arrest and DNA damage-inducible protein; GLS2: glutaminase 2; GPx1: glutathione peroxidase-1; IGF-1: insulin-like growth factor 1; MDM2: mouse double minute 2 homolog; MDMX: double minute X human homolog; MDR1: multiple drug resistance gene 1; Noxa: damage protein, a proapoptotic BH3-containing protein; p21: cyclin-dependent kinase inhibitor; p53: tumor suppressor; PCNA: proliferating cell nuclear antigen; PERP: p53 apoptosis effector related to PMP22; Pml: promyelocytic leukemia protein; Puma: p53 upregulated modulator of apoptosis; TERT: telomerase reverse transcriptase; TIGAR: TP53-inducible glycolysis and apoptosis regulator; Ypel3: Yippee-like 3.

In response to a broad range of stresses, p53 upregulates and enhances its expression to perform a suitable cellular response. Oncogenic signaling produces the alternate reading frame (ARF), tumor suppressor, to inhibit the expression of MDM2 and MDMX [91, 92]. Recent research has also been associated with the p53 via the MDM2-ARF pathway leading to cell cycle arrest and apoptosis [19, 93]. DNA damage activates p53 through ATM and ATR protein kinase-dependent pathways [94]. Once ATM and ATR are activated in response to DNA damage, p53 is phosphorylated, resulting in p53 uncoupling from the E3 ubiquitin ligase MDM2 proto-oncogene (MDM2) and negative regulator MDMX, leading to p53 stability and activation. [95]. ATM and ATR are large kinases with sequence similarity to lipid kinases of the phosphatidylinositol 3-kinase (PI3K) family that phosphorylate protein substrates [96]. Chk1 and Chk2 kinases are selectively phosphorylated and activated by ATR and ATM regulators, respectively [97]. The p53 pathways respond to different aberrant DNA structures. ATM is activated primarily at DNA double-strand breaks (DSBs) with the MRE11-RAD50-NBS1 (MRN) complex [98], and ATR is activated at single-strand DNA (ssDNA) with ATR-interacting protein (ATRIP) [99] (Figure 2).

ATM monomers are then recruited to DSBs with the MRN sensor complex stimulating the activation on multiple substrates at the site of damage [100]. Substrates include the variant histone H2AX, forming the DNA damage-associated *γ*H2AX histone mark in the MRN complex itself, and the downstream effector kinase Chk2 [101]. ATM phosphorylates Chk2, leading to transient homodimerization, intermolecular activation loop autophosphorylation, and full activation [102, 103]. Chk2 dissociates from the site of damage and disperses a monomer to act on multiple substrates involved in cell cycle progression, apoptosis, and gene transcription [104]. Chk2 substrates include p53 [105] and its regulator MDMX [106]. Other substrates targeted by ATM at sites of damage include NBS1, BRCA1, MDC1, and p53BP1 [107]. ATM plays an essential role in the activation of the p53 response to DNA damage by phosphorylating p53 itself and the stability regulators such as MDM2 and MDMX [108].

ATR-Chk1 signaling is activated strongly when DNA replication is impeded. DNA polymerases become uncoupled from the replicative helicase, generating tracts of ssDNA that rapidly become coated with the trimeric ssDNA-binding protein complex replication protein A (RPA) [109]. ATR is activated in association with its partner protein ATRIP which interacts with ssDNA complexed with RPA via the 70 kDa RPA1 subunit [110]. ATR activation and Chk1 phosphorylation depend on the action of two mediator proteins, TopBP1 and Claspin. TopBP1, which is recruited to ssDNA-RPA via the PCNA-like RAD9:RAD1:HUS1 checkpoint clamp, contains a domain that stimulates ATR activity [111, 112]. Claspin likely relates to active replication forks when normal replication is subjected to ATR-dependent phosphorylation, which modified and bound Chk1 to serve as a platform for ATR-mediated phosphorylation and activation [113].

ATR-Chk1 and ATM-Chk2 signaling are known for the stabilization and phosphorylation of p53. Chk1 and Chk2 activities are required for p53 phosphorylation [114]. p53 exerts antioxidant activity depending on the extent of stress signals. p53 induces a group of antioxidant genes, including sestrin 1/2, TP53-inducible glycolysis and apoptosis regulator (TIGAR), glutathione peroxidase-1 (GPx1), aldehyde dehydrogenases (ALDH4), glutaminase 2 (GLS2), and Parkin, especially under nonstress or low-stress conditions, to lower ROS levels and to repair the DNA successfully. This antioxidant activity protects cells from oxidative stress-induced DNA damage and mutations and promotes cell survival [115]. Growth arrest and DNA damage-inducible proteins such as GADD45, p21, Ypel3, and Pml are induced by p53 and lead to cell cycle arrest. p53-mediated apoptosis induced by the apoptotic genes, including DR5, Fas, PERP, Bcl-2, Bax, Noxa, and Puma, which takes place in intrinsic and extrinsic pathways, leads to apoptosis in case of failure to repair DNA [30, 116]. If the p53 gene is damaged, tumor suppression is severely reduced. Mutant p53 transactivates several genes, including MDR1, c-myc, PCNA, IL-6, IGF-1, FGF, EGFR, ASNS, and TERT. Mutant p53 promotes oncogenic genes, which lead to the progression of aggressive cancers [90].

### 5.1. Regulation of p53 by Posttranslational Modifications

p53 is modulated by a few fundamental processes: acetylation, phosphorylation, ubiquitination, sumoylation, neddylation, and methylation [116]. p53 undergoes a sequence of posttranslational modifications, mainly due to DNA damage, oncogene activation, and hypoxia. The posttranslational modification in p53 occurs by acetylation and phosphorylation, which leads to tetramerization to reach functional stability and reverse the effects of cellular and genotoxic insults [26, 116].

#### 5.1.1. Phosphorylation

Phosphorylation responses to DNA damage and other stresses are controlled by regulators, subcellular localization, stability, and transcriptional activation of target genes, which prompts the biological effects [116, 117]. Several kinases target specific serine and threonine residues within p53 for phosphorylation [118]. ATM, ATR, and DNA-dependent protein kinase (DNA-PK) are activated by DNA damage and directly phosphorylate Ser15, Ser20, and Ser46 of p53 [76]. Ser15 is targeted by ATR, DNA-PK, and Chk1, whereas Ser20 of p53 is a target in Chk2 kinase [119–121]. Chk1 and Chk2 can also phosphorylate more p53 residues, such as Thr387 by Chk1, Thr18, and Ser366 by Chk2, and also 4 residues, namely, Ser313, Ser314, Thr377, and Ser378, in the C-terminus by both kinases [122]. Ser46 is the major phosphorylation of p53 by several kinases required for apoptosis in response to DNA damage, and increased Ser46 phosphorylation of p53 induces apoptotic target gene transcription [123]. Changes in phosphorylation of p53 either facilitate or inhibit the binding of specific interactors to p53 that can block or promote acetylation [116].

#### 5.1.2. Acetylation

Acetylation of p53 is another mechanism to stabilize the p53 protein that reduces ubiquitination and degradation. SIRT1 plays an important role by deacetylating p53 protein [124]. Acetylation of p53 blocks the ubiquitin sites and prevents ubiquitination by MDM2 and subsequent degradation [125]. Acetylation also recruits p300/CBP/PCAF to the promoter regions for activation of p53-targeted genes, such as p21, MDM2, and Puma [126]. Conversely, loss of eight major acetylation sites in human p53, namely, Lys164, Lys305, Lys320, Lys372, Lys373, Lys381, Lys382, and Lys386, renders p53 transcriptionally and prevents its induction of cell cycle arrest and apoptosis [127]. Six C-terminal residues such as Lys305, Lys372, Lys373, Lys381, Lys382, and Lys386 and one DBD residue Lys164 are acetylated by CBP/p300; however, Lys320 is acetylated by PCAF [126–128]. Histone deacetylase (HDAC) inhibitors are also reported, which induce acetylation in nonhistone proteins [129]. Inhibition of HDAC eliminates HDAC1 and SIRT1 from p53, which increases p53 acetylation and p53-dependent activation of apoptosis and senescence [71].

### 5.2. Regulation of Autophagy by p53

Autophagy is the mechanism of cells for eliminating unnecessary or dysfunctional cellular components. p53 signaling regulates autophagy, and p53 is an autophagy target protein. Under nutrient starvation and various stressful conditions, such as hypoxia, both the wild-type and mutant p53 can be degraded through autophagy [130–132]. As a result, autophagy is often found in tumor cells that have been subjected to chemotherapy or radiation [133]. In tumor cell proliferation, cell cycle control, apoptosis, senescence, and autophagy, p53 plays a regulatory role. The subcellular localization of p53 is linked to its regulatory function. When p53 is localized in the nucleus, it encourages autophagy in stressed cells, while cytosolic p53 prevents autophagy in nonstressed cells [134]. p53 induces autophagy in the nucleus by transcriptionally controlling the mammalian target of rapamycin (mTOR) pathway as well as transcriptional regulation of key autophagy-related genes (ATGs) [135]. The phosphatase and tensin homolog (PTEN), tuberous sclerosis-2 (TSC2), and AMP-activated protein kinase (AMPK) are some of the p53-targeted genes that have been shown to inhibit mTOR, facilitating autophagy initiation. Furthermore, the tumor suppressor protein p14 ADP-ribosylation factor (ARF) signaling pathway has been linked to p53 and autophagy [136]. Activation of oncogenes during tumorigenesis increases the transcription of ARF, which binds to and inhibits the expression of MDM2, stabilizing p53 [137]. By disrupting the Bcl-xl/Beclin 1 complex and releasing Beclin 1 to induce autophagy, ARF promotes autophagy [138]. Autophagy inhibition with chemical inhibitors or downregulation of the basic autophagy genes ATG1/ULK1, Beclin 1, or ATG5 results in the stabilization of mutant p53 under basal growth conditions [139, 140].

p53 can also mediate functional responses between apoptosis and autophagy when it is subcellularly localized. p53 can localize to mitochondria, where it interacts with antiapoptotic B-cell lymphoma (Bcl) family proteins, allowing proapoptotic factors like Bcl-2-associated X protein (Bax) and Bcl-2-antagonist/killer (BAK) to oligomerize, facilitating mitochondrial outer membrane permeabilization and driving activation of the intrinsic apoptotic cell death pathway [141, 142]. Beclin 1 and ATG7, autophagy-related proteins, avoid mutant p53 degradation caused by turmeric or curcumin [143, 144]. In another study, MDA-MB-231, MDA-MB-468, T47D, and SKBr3 breast cancer cell lines expressing different mutant p53 failed to form complexes with Bcl-2 when compared to human acute myeloblastic leukemia ML-1 cells [145]. On the other hand, overexpression of Beclin 1 or ATG1/ULK1 causes mutant p53 depletion. In MDA-MB-231 and DLD1 cancer cell lines, gambogic acid, a molecule that stimulates the degradation of mutant p53, can induce the degradation of mutant p53 proteins through autophagy [146]. Autophagy inhibition with bafilomycin A1 or chloroquine prevents gambogic acid from degrading mutant p53. Glucose restriction causes mutant p53 deacetylation, which directs it to autophagy for degradation [147]. Depletion of p53 causes autophagy activation and cell death, while the expression of a degradation-defective mutant p53 prevents autophagy and allows survival in the face of glucose restriction. A novel curcumin-based zinc compound has been found to cause mutant p53 protein degradation via autophagy [148].

## 6. p53 Signaling Pathway Modulated by Dietary Antioxidants

Numerous *in vitro* and *in vivo* studies have demonstrated that dietary biologically active (bioactive) compounds can modulate immunity to delay cancer development and progression through decreased cell proliferation, inactivation of carcinogens, inhibition of angiogenesis, induction of cell cycle arrest, apoptosis, and regulation of various signaling pathways [19, 149–152]. Chemopreventive and anticancer effects of dietary antioxidants have been reported from several normal cell lines, cancer cell lines, and animal studies by the p53 signaling pathway (Table 1).

### 6.1. Vitamins

*In vitro* experiments have shown that vitamin C (ascorbic acid) can reduce cell proliferation and induce apoptosis through upregulation of p53, p21, and Bax and downregulation of Bcl-2 in T-cell colonies [153]. Furthermore, Harakeh and colleagues demonstrated that the administration of nontoxic doses of ascorbic acid increased the expression of p53 [153]. Vitamin C increases the ability of the anticancer drug bleomycin to produce DSBs, which makes cancer cells more dependent on functional DNA repair for survival [99]. Vitamin B_6_ activates the p53 pathway, which is responsible for controlling p21 mRNA transcription in HT29, Caco2, LoVo, HEK293T, and HepG2 cancer cells. p21 mRNA levels were higher in the colon of mice fed a diet with adequate vitamin B_6_ than those fed a vitamin B_6_-deficient diet, and this may help to understand the antitumor effect of vitamin B_6_ via the activation of p53 and elevation of p21 mRNA [154]. A previous study suggested that 1,25-dihydroxyvitamin D increased oxidative stress through inhibiting transcription of Nrf2, enhancing DNA damage and activation of p16/Rb and p53/p21 signaling in a 1*α*(OH)ase−/− mouse model [155]. Folic acid (vitamin B_9_) might play an important role in the chemoprevention of gastric carcinogenesis. In humans, the tumor suppressor p53 expression in the gastric mucosa was significantly increased, while the expression of Bcl-2 oncogene protein decreased after folic acid supplementation [156]. Furthermore, N-acetylcysteine (NAC) inhibits PDK1 expression through PPAR*α*-mediated induction of p53 and reduction of p65 protein expression and unveils a novel mechanism by which NAC in combination with the PPAR*α* ligand inhibits the growth of non-small-cell lung carcinoma (NSCLC) cells [157]. Water-soluble vitamin E (Trolox) supplementation promoted breast cancer growth by reducing ROS and p53 expression in mice [158]. *β*-Carotene, ascorbic acid, and vitamin E (*α*-tocopherol) protect against oxidative stress but reveal no direct influence on p53 expression in rats subjected to stress [159]. In contrast, *β*-carotene exacerbates DNA oxidative damage and modifies p53-related pathways of cell proliferation and apoptosis in cultured RAT-1 fibroblasts exposed to tobacco smoke condensate (tar) [160].

### 6.2. Flavonoids

Flavonoids are suggested to reduce p53-mediated chronic inflammation leading to cancer progression [161]. Quercetin can induce phosphorylation of ATM and phosphorylation of histone H2AX; therefore, p53 accumulation and phosphorylation occurred in ATM-deficient cells, indicating that ATM is not required for quercetin-induced p53 phosphorylation [162]. DNA lesions are produced by quercetin, and the mechanisms to detect this damage must be subtly different from the ability of quercetin to induce ATM-independent p53 phosphorylation [162]. Quercetin increased the phosphorylation of p53 protein and induced apoptosis of the human leukemia cell line in a dose-dependent manner [163]. A recent study revealed that quercetin inhibits HeLa cell proliferation through cell cycle arrest at the G_2_/M phase and apoptosis induction through the disruption of mitochondrial membrane potential and activation of the intrinsic apoptotic pathway through p53 induction [164]. Further, apigenin can induce p21, p53, and nonsteroidal anti-inflammatory drug-activated gene-1 (NAG-1) proteins in kinase pathways, including protein kinase C delta (PKCd) and ATM, which plays an important role in activating these proteins in colorectal cancer cell growth arrest [165]. Further, kaempferol warrants as an antiangiogenetic agent, which reduced human umbilical vein endothelial cell viability-induced DNA damage and DNA fragmentation through activating the levels of caspase-3, caspase-8, and caspase-9 signaling, which were upregulated by ROS-mediated p53/ATM molecules following stimulations of p53 downstream protein levels of Fas/CD95, death receptor 4 (DR4), and DR5 [166]. Another study revealed acacetin, an O-methylated flavone, which can strongly inhibit tumor growth and induce tumor shrinkage in mice, which is closely correlated with its increasing p53 expression accompanied by decreased retinoic acid receptor gamma (RAR*γ*) and reduced AKT activity in liver cancer cell lines [167]. It was further reported that securin and p53 play an important role in determining the sensitivity of human colon cancer cells to fisetin. Depletion of securin enhances fisetin-induced apoptosis and decreases the resistance of p53-deficient cells to fisetin and might be an attractive strategy for the treatment of human colon cancers [168]. The inhibitory effect of fisetin against bladder cancer by activation of p53 and downregulation of the nuclear factor-kappa B (NF-*κ*B) pathway in a rat bladder carcinogenesis model has been documented, which is a safe and efficacious agent and promising therapeutic approach for bladder cancer [169]. Furthermore, luteolin treatment increases the expression of p53 and p21 proteins and decreases the expression of MDM4 protein in both NSCLC cells and tumor tissues [170]. Theaflavins induced G_2_/M arrest by modulating the expression of various proteins, which are involved in signaling. Moreover, theaflavins via p53 signaling inhibited Bcl-2 and interfered phagocytes via modulation of I-*κ*B/NF-*κ*B, as well as the expression of VEGF, and the phosphorylation of VEGFR was reduced in LNCaP cells [171]. Furthermore, epigallocatechin-3-gallate activates p53-dependent downstream targets p21/WAF1 and Bax and downregulates NF-*κ*B-dependent Bcl-2 that results in growth arrest and apoptosis in LNCaP cells [93]. Our previous study revealed that effector proteins like Chk1, Chk2, and p53 were found to be phosphorylated in NNK acetate-treated BEAS-2B cells, and pretreatment with apple flavonoids showed a significant reduction in the levels of phosphorylation of ATR, Chk1, and p53 in NNK acetate-treated cells [172]. Apple flavonoids protect BEAS-2B cells challenged against various carcinogens by assisting DNA repair mechanisms [172]. Further, targeted drug delivery of encapsulated C3G chitosan nanoparticles can downregulate p53-mediated apoptosis in Kunming mice, which provides the powerful photochemopreventive effect [173].

### 6.3. Phenolic Acids

Caffeic acid increases p53 protein expression in a concentration-dependent manner and induces apoptosis by inhibiting Bcl-2 activity, leading to the release of Cyt c and subsequent activation of caspase-3 in HeLa cells [174]. Pretreatment of caffeic acid with a p38 MAPK specific inhibitor significantly inhibited p53 phosphorylation at serine 15. However, other MAPK-mediated p53 phosphorylation sites might be activated by CAPE, and the effect of CAPE activated ERKs in C6 glioma cells [175]. A study provided evidence that ellagic acid inhibited cell proliferation via p21-mediated G_1_ arrest and cell death, leading to inhibition of tumor cell proliferation and induction of cell death by ellagic acid, which supports as a chemopreventive agent [176].

### 6.4. Other Polyphenols

Curcumin increases levels of p53, which provides an appropriate cellular environment for p53 and NAD(P)H quinone oxidoreductase (NQO1) to interact in HeLa, SiHa, and CaSki tumor-derived cell lines. At the same time, this interaction promotes the loss of interaction of p53 with its negative regulator E6AP ubiquitin-protein ligase (E6AP) when human papillomavirus E6 (HPV E6) oncoproteins are present [177]. Another study suggested that upregulation of both p53 and p21 in U266 cells exposed to curcumin is involved in cell cycle arrest [178]. Furthermore, curcumin decreased the expression of p53 and p21 in Wistar rats [179]. Resveratrol metabolites can promote a moderate cellular senescence induction in breast cancer cells, which involves inhibiting cell growth by G_2_/M phase arrest through the p53/p21Cip1/WAF1 pathway [180]. A previous study has revealed that SET7/9 functions as a mediator of resveratrol-dependent p53 activation, and the results confirm that resveratrol modulates p53 through its monomethylation at K372 [181]. Another study investigated the effect of red wine on anticancer and chemopreventive properties. Polyphenol-rich red wine extracted from grapes could increase the active phosphorylated p53 in human lung cancer cells [182].

### 6.5. Alkaloids

Caffeine suppresses the ATM/p53 signaling pathway in human T-lymphocyte leukemia MOLT-4 cells. Caffeine suppressed p21 upregulation and inhibited p53 phosphorylation on Ser15 and Ser392, which is ATM-independent, suggesting that caffeine might have another cellular target [183]. Caffeine inhibits *γ* and UV radiation-induced phosphorylation of p53 and Ser15, which may be modified directly mediated by ATM [183]. Furthermore, Kaufmann et al. [184], in their work on human fibroblasts, described the ATM-dependent phosphorylation of p53 after *γ* irradiation as resistant to caffeine. Evidence indicated that harmine treatment induced apoptosis in MCF-7 cells through the upregulation of p53 expression and reported that harmine is able to upregulate p53 gene expression in breast cancer cell lines. Furthermore, harmine induced apoptosis in breast cancer cells in a time-dependent manner and real-time PCR outcomes on breast cancer cells showed that apoptosis induction in MCF-7 cells by harmine requires the activation of p53 gene expression [185]. Colchicine exhibits *in vitro* antiproliferative activity against HPV 16/18-positive human cervical cancer cell lines, which was achieved by HPV E6/E7 inhibition and subsequently p53-dependent intrinsic apoptosis [186].

## 7. Dietary Antioxidants as Anticancer Agents

Dietary antioxidants, including polyphenols and vitamins, have been investigated for their prospective benefits such as antioxidant, antiproliferative, antiangiogenic, anticancer, immunomodulatory, and hypoglycemic properties, which increases their applications in the pharmaceutical, functional food, and cosmetic industries [10, 187, 188]. The anticancer potential of dietary antioxidants was well addressed due to their wide distribution in nature, which includes a large number of dietary sources extracted from plants as well as isolated as pure compounds [189–192]. Moreover, several therapeutic modifications have been employed to get better outputs, such as the application of nanotechnologies to increase the solubility and bioavailability of polyphenols [193, 194]. However, there are several pieces of evidence suggesting that p53 mediated apoptosis by dietary antioxidants, which leads to cancer growth inhibition and prevention. When p53 is mutated or absent, the dietary antioxidants exert an antitumor property, and the cells are resistant to current therapies [19]. Numerous researchers have demonstrated that anticarcinogenic effects of polyphenols are assigned to several mechanisms, including the induction of apoptosis, regulation of various cell signaling pathways, regulation of the cell cycle, DNA repair, and activation of the receptors [195, 196]. Flavonoids are known to activate the Keap1/Nrf2/ARE pathway and contribute to cytoprotection in normal cells [197]; however, some flavonoids such as flavones, luteolin, apigenin, and chrysin are shown to inhibit Keap1/Nrf2/ARE and are proapoptotic in cancer cells [198].

## 8. Clinical Studies

Anticancer effects of dietary antioxidants targeting the p53 signaling pathway have been identified using several cell-based and experimental animal models [19]. In humans, folic acid supplementation significantly increased p53 expression in the gastric mucosa [156]. Epigallocatechin-3-gallate (EGCG) consumption causes apoptosis in human prostate carcinoma cells by phosphorylating essential serine residues on p53, resulting in upregulation of its transcriptional function [93]. Naringenin has been shown to inhibit cell proliferation and cause apoptotic cell death in human hepatocellular carcinoma, making it a potential candidate for liver cancer therapy [199]. In human hepatocellular carcinoma cells, naringenin treatment causes G_0_/G_1_ and G_2_/M phase arrests, which may be due to rapid p53 accumulation [200]. Quercetin has also been shown to prevent human breast cancer cells from entering the S phase by increasing the levels of the proteins of p53 [201]. A preclinical animal model of skin cancer has demonstrated the synergistic effects of resveratrol and black tea polyphenols in suppressing the development of skin tumors. Treatment with this combination of dietary antioxidants causes a decrease in the expression of phosphorylated mitogen-activated protein kinase (MAPK) family proteins, extracellular signal-regulated kinase 1/2, c-Jun N-terminal kinase, and p38, as well as an increase in total p53 and p-p53, which shows promise in suppressing the growth of mouse skin tumors [202]. Using *in vitro* and *in vivo* models of research, the synergistic effects of the green tea extract EGCG and tisane herbal infusion in the eradication of malignant human prostate tumors were investigated. Apoptotic genes such as p53, p73, p21, and caspase-3 were all upregulated as a result of the antioxidant combination [203]. Intravesical fisetin has been shown to inhibit bladder cancer in a rat bladder carcinogenesis model by activating p53 and downregulating the NF-*κ*B pathway. It has been proposed that intravesical fisetin is a novel and promising therapeutic solution for bladder cancer because it is a safe and effective therapeutic agent [169].

## 9. Conclusion and Future Direction

Cancer is one of the most common causes of disease worldwide. Currently, chemotherapy and radiotherapy are two major cancer treatments, although both are associated with prominent side effects. It is necessary to identify efficacious alternative pharmaceuticals with selective cytotoxicity to cancer cells with the aim of reducing the side effects. Also, novel approaches need to be investigated to reduce the risk of various cancers. Diets rich in polyphenols are implicated in protecting against oxidative stress and inflammation-mediated chronic diseases such as cardiovascular diseases, neurodegenerative disorders, and certain cancers. The cancer chemopreventive effects of dietary polyphenols and other antioxidants are well documented. Dietary polyphenols are able to modulate multiple cellular pathways, which are involved in the initiation and progression of cancer. Polyphenols found in fruits, vegetables, herbs, wine, and teas are safe for consumption. Thus, dietary polyphenols offer multitargeted therapeutic effects than a single drug. The chemopreventive effects of polyphenols have been shown through the modulation of various signaling pathways, including the p53 pathway. p53 signaling activates transcription factors in response to oxidative stresses. In this review, we have shown that certain polyphenols can stabilize p53 protein via phosphorylation and acetylation, contributing to oxidative homeostasis. p53 binds within the DNA and regulates transcriptional genes involved in DNA repair, metabolism, senescence, apoptosis, autophagy, and angiogenesis. Future studies need to be aimed at investigating the efficacy of specific dietary polyphenols and other natural antioxidants and their mixtures against various carcinogen-induced DNA damage through the activation of p53 in healthy cells using *in vitro* models, preclinical experimental animal models, and human studies.

## Figures and Tables

**Table 1:** Dietary antioxidants show the expression of p53 in experimental models.

Antioxidants	Experimental model	Mechanism	Expression of p53	References
(i) Cancer cells				
Vitamin C	T-lymphocyte cell lines (HuT-102, C91-PL, CEM, and Jurkat)	p53 ↑, p21 ↑, Bax ↑, Bcl-2 ↓	Protein	[153]
	Human lung carcinoma cell lines (H460 and A549)	p53 ↑, caspase-3 ↑, caspase-7 ↑	Protein, mRNA	[99]
Vitamin B_6_	HT29, Caco2, LoVo, HEK293T, and HepG2 cell lines and ICR mice	p53 ↑, p21 ↑	Protein, mRNA	[154]
Vitamin D	Mice (1*α*(OH)ase−/−)	p53 ↑, p21 ↓, p16 ↑	Protein, mRNA	[155]
Folic acid	Human	p53 ↑, Bcl-2 ↓	Protein	[156]
N-Acetylcysteine	Non-small-cell lung carcinoma (NSCLC) cell line	p53 ↑, p65 ↓	Protein, mRNA	[157]
Vitamin E	Human breast cancer cell line (MCF-7)	p53 ↓	Protein, mRNA	[158]
*β*-Carotene	RAT-1 murine fibroblast cell line	p53 ↑, p21 ↑, Bax ↑, Bcl-2 ↓	Protein	[160]
Quercetin	Human cervical cancer cell line (HeLa)	p53 ↑, caspase-3 ↑, caspase-9 ↑, Bax ↑, Bcl-2 ↓	Protein, mRNA	[164]
Apigenin	Human colon cancer cell lines (HCT116 and HT29)	p53 ↑, p21 ↑, NAG-1 ↑	Protein, mRNA	[165]
Acacetin	Human liver cancer cell line (HepG2)	p53 ↑, Bax ↑	Protein, mRNA	[167]
Fisetin	Human colon cancer cell line (HCT116)	p53 ↑, caspase-3 ↑	Protein, mRNA	[168]
Luteolin	Human lung cancer cell lines (A549 and H460)	p53 ↑, p21 ↑, MDM4 ↓	Protein, mRNA	[170]
Theaflavin	Human prostate cancer cell line (LNCaP)	p53 ↑, Bcl-2 ↓, Bax ↑	Protein	[171]
Epigallocatechin-3-gallate (EGCG)	Human prostate cancer cell line (LNCaP)	p53 ↑, p21 ↑, Bcl-2 ↓, Bax ↑, caspase-3 ↑, caspase-8 ↑, caspase-9 ↑	Protein	[93]
Caffeic acid	Human cervical cancer cell line (HeLa)	p53 ↑, Bcl-2 ↓	Protein	[174]
Caffeic acid phenethyl ester	C6 glioma cell line	p53 ↑, Bcl-2 ↓, Bax ↓	Protein	[175]
Ellagic acid	Human cervical cancer cell line (CaSki)	p53 ↑, p21 ↑, GAPDH ↑	Protein, mRNA	[176]
Curcumin	Human cervical cancer cell lines (HeLa, SiHa, and CaSki)	p53 ↑	Protein	[177]
	Multiple myeloma cell line (U266B1)	p53 ↑, p21 ↑	Protein	[178]
Resveratrol	Human breast cancer cell line (MCF-7)	p53 ↑, p21 ↑, p16 ↑	Protein	[180]
	Human colon cancer cell lines (HCT116, CO-115, and SW48)	p53 ↑, PARP ↓, caspase-3 ↓	Protein	[181]
Red wine	Human lung cancer cell line (A549)	p53 ↑, p53 (Ser15) ↑	Protein	[182]
Caffeine	Human T-lymphocyte leukemia MOLT-4 cell line	p53 ↑, p21 ↑, Mcl-1 ↓	Protein	[183]
Harmine	Human breast cancer cell line (MCF-7)	p53 ↑	mRNA	[185]
Colchicine	Human cervical cancer cell lines (CaSki and HeLa)	p53 ↑, Bcl-2 ↓, Bax ↑	Protein, mRNA	[186]
(ii) Normal cells				
*α*-Tocopherol, ascorbic acid, & *β*-carotene	Rat	p53 ↓, MDM2 ↓	Protein	[159]
Kaempferol	Human umbilical vein endothelial cell line (HUVEC)	p53 ↑, ATM ↑, Fas ↑, DR5 ↑, caspase-3 ↑, caspase-8 ↑, caspase-9 ↑	Protein	[166]
Fisetin	Wistar rat	p53 ↓, p21 ↓, Bax ↓, Bcl-2 ↑	Protein	[169]
Apple flavonoids	Normal human bronchial epithelial cell line (BEAS-2B)	p53 ↓, Chk1 ↓, ATR ↓	Protein	[172]
Cyanidin-3-*O*-glucoside (C3G)	Kunming mice (KM)	p53 ↓, Bax ↓, caspase-3 ↓, caspase-9 ↓, Bcl-2 ↑	Protein	[173]
Curcumin	Wistar rat	p53 ↓, p21 ↓	Protein	[179]
Caffeine	Normal human fibroblast cell line (NHF1)	p53 ↓, p21 ↓	Protein	[184]

## Abbreviations

ATM: Ataxia telangiectasia-mutated protein kinase
ATR: ATM-Rad3-related protein kinase
Bax: Bcl-2-associated X protein
BID: BH3 domain-only death agonist protein
BRCA1: Breast cancer gene 1
Chk1: Checkpoint kinase 1
Chk2: Checkpoint kinase 2
Cyt c: Cytochrome c
DDR: DNA damage response
DNA: Deoxyribonucleic acid
DR5: Death receptor 5
DSBs: Double-strand breaks
Fas: Fas cell surface death receptor
MDM2: Mouse double minute 2 homolog
MDMX: Double minute X human homolog
MRN: MRE11-RAD50-NBS1
p21: Cyclin-dependent kinase inhibitor
p53: Tumor suppressor
PERP: p53 apoptosis effector related to PMP22
ROS: Reactive oxygen species
RPA: Replication protein A
ssDNA: Single-strand DNA.
